# Serum malondialdehyde predicts mortality in patients with acute heart failure

**DOI:** 10.1016/j.redox.2026.104129

**Published:** 2026-03-15

**Authors:** Marija Pinterić, Iva Klobučar, Margarete Lechleitner, Lidija Hofmann, Matias Trbušić, Gudrun Pregartner, Andrea Berghold, Tobias Madl, Saša Frank, Vesna Degoricija

**Affiliations:** a Gottfried Schatz Research Centre, Division of Molecular Biology and Biochemistry, Medical University of Graz, Graz, 8010, Austria; b Department of Cardiology, Sisters of Charity University Hospital Centre, Zagreb, 10000, Croatia; c Institute of Biomedical Science, Department of Health Studies, FH JOANNEUM University of Applied Sciences, Graz, 8020, Austria; d University of Zagreb School of Medicine, Zagreb, Croatia; e Institute for Medical Informatics, Statistics und Documentation, Medical University of Graz, Graz, 8036, Austria; f Otto Loewi Research Centre, Division of Medicinal Chemistry, Medical University of Graz, Graz, 8010, Austria; g BioTechMed-Graz, Graz, 8010, Austria; h Department of Medicine, Sisters of Charity University Hospital Centre, Zagreb, 10000, Croatia

**Keywords:** Malondialdehyde, Catalase, Superoxide dismutase, Acute heart failure, Mortality, Oxidative stress

## Abstract

**Aims:**

Acute heart failure (AHF) is associated with high short- and long-term mortality, and early identification of patients at highest risk remains challenging despite the use of established biomarkers and clinical risk scores. Oxidative stress plays a central role in the pathophysiology of AHF but has been insufficiently investigated as a prognostic target. This study aimed to evaluate the prognostic value of serum oxidative stress biomarkers for predicting short- and long-term mortality in AHF patients and to determine whether they improve risk stratification beyond established tools.

**Methods:**

Admission serum levels of malondialdehyde (MDA), advanced oxidation protein products (AOPPs), catalase, and superoxide dismutase (SOD) were measured in a cohort of 315 hospitalized AHF patients. Univariable and multivariable logistic regression analyses were performed to assess associations with in-hospital, 6-month, and 12-month mortality. Prognostic performance was compared with N-terminal pro-brain natriuretic peptide (NT-proBNP) and established risk scores (ADHERE, GWTG-HF, OPTIMIZE-HF) using receiver operating characteristics curves, the continuous net reclassification index (cNRI) and decision curve analysis.

**Results:**

Serum MDA, catalase, and SOD were significantly elevated in non-survivors at all time points. In the adjusted analyses, MDA was the only oxidative stress marker independently associated with mortality. MDA showed robust prognostic performance, with improvements in AUC and cNRI as well as increased net benefit, when added to NT-proBNP or clinical scores. Survival analysis confirmed lower survival in patients with MDA ≥1.45 nmol/L (the median in our cohort). Other biomarkers (AOPPs, catalase, SOD) showed limited prognostic utility.

**Conclusion:**

Serum MDA is a robust, independent predictor of mortality in AHF. Incorporating MDA into routine assessment may enhance early identification of high-risk patients and support personalized management strategies in AHF.

## Highlights

• We evaluated the prognostic value of MDA, AOPPs, catalase, and SOD in patients with AHF.
• Only MDA independently predicted short- and long-term mortality in AHF.
• Adding MDA to NT-proBNP and clinical risk scores improved discrimination for mortality.
• High MDA levels were linked to poor survival in patients with AHF.

## Graphical abstract

**Figure:**
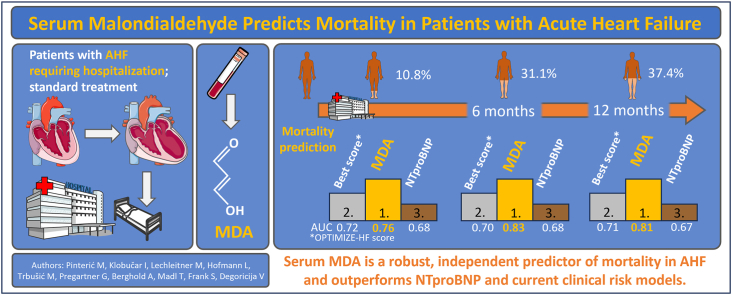

## 1 Introduction

Heart failure (HF) affects over 26 million people worldwide and remains a leading cause of morbidity, hospitalization, and mortality [1]. Among its forms, acute heart failure (AHF) is particularly severe, often requiring urgent intervention, yet early identification of patients at highest risk remains challenging despite established biomarkers and clinical scores [2].

Oxidative stress, resulting from an imbalance between reactive oxygen species (ROS) production and antioxidant defenses, is central to the HF pathophysiology [3]. Excess ROS contributes to myocardial injury, fibrosis, endothelial dysfunction, and apoptosis, thereby promoting progressive cardiac dysfunction [4,5]. In HF, multiple mechanisms, including hemodynamic and metabolic disturbances, neurohormonal activation, and systemic inflammation, further amplify oxidative damage [3,6,7].

Oxidative stress biomarkers such as malondialdehyde (MDA) and advanced oxidation protein products (AOPPs) reflect lipid and protein oxidation, respectively, whereas catalase and superoxide dismutase (SOD) indicate the activity of endogenous antioxidant defense systems [5,7,8]. Although previous studies have linked oxidative stress biomarkers with outcomes in chronic HF [[9], [10], [11], [12], [13]], their prognostic utility and incremental value beyond established biomarkers in AHF remain insufficiently explored.

To fill this gap, the present study aimed to evaluate the prognostic relevance of MDA, AOPPs, catalase, and SOD in patients with AHF, assessing whether these markers improve risk stratification beyond established biomarkers and clinical risk models.

## 2 Methods

### 2.1 Study cohort

This prospective, observational study enrolled adult patients who were urgently hospitalized due to AHF at the Sisters of Charity University Hospital Centre in Zagreb, Croatia, between 2018 and 2021. Diagnosis and treatment of AHF followed the 2016 European Society of Cardiology Heart Failure Guidelines, which were valid at the time of enrollment [14]. Detailed inclusion and exclusion criteria, study protocols, blood collection and routine laboratory analyses as well as the patient flow chart have been previously published [15]. In brief, medical history and physical examination data were collected at presentation to the emergency department, and venous blood samples were collected in 6 mL tubes with a VACUETTER® Z Serum Clot Activator (Greiner Bio-one GmbH, Kremsmuenster, Austria) prior to the initiation of any pharmacologic treatment. Echocardiography was performed within the first 24 h of hospitalization. The primary study outcomes were in-hospital, 6-month, and 12-month all-cause mortality. All participants provided written informed consent prior to any study-related procedure. The study was approved by the Ethics Committee of the Sisters of Charity University Hospital Centre, Zagreb, Croatia (EP 2258/18-10), and the Ethics Committee of the Medical University of Graz, Austria (EK 33-258 ex 20/21). The study was conducted in accordance with the principles of Good Clinical Practice and the Declaration of Helsinki [16].

### 2.2 Preparation and handling of serum

Serum was prepared by incubation of collected blood samples at room temperature for 30 min, followed by centrifugation at 1800×*g* for 10 min at 4 °C. The resulting serum was portioned into 1 mL aliquots and stored at −80 °C. For subsequent analyses, the aliquots were thawed on ice and transferred into pre-cooled tubes in volumes suited to each analytical technique before being returned to −80 °C for storage until analysis.

### 2.3 Quantification of serum MDA levels

MDA levels were quantified using a modified thiobarbituric acid (TBA) reactive substances assay [17,18]. To minimize non-specific signals and measure free MDA, 200 μL of serum was deproteinized by adding an equal volume (200 μL) of 1 M perchloric acid (PCA; Sigma-Aldrich, St. Louis, MO, USA). The mixture was vortexed, incubated at room temperature for 5 min, and centrifuged at 12,000×*g* for 4 min. Supernatants (150 μL, in duplicate) were mixed with 150 μL of 2% sodium dodecyl sulfate (SDS; Carl Roth GmbH + Co. KG, Karlsruhe, Germany), followed by the addition of 380 μL of 28.5 mM TBA (Sigma-Aldrich, St. Luis, MO, USA) prepared in a 1:1 mixture of water and acetic acid, adjusted to pH 3.5. Samples were incubated at 95 °C for 1 h in a preheated sand (Carl Roth GmbH + Co. KG, Karlsruhe, Germany) bath, then rapidly cooled on ice, vortexed, and centrifuged. A 200 μL aliquot from each reaction mixture was transferred, in duplicate, to a 96-well plate, and absorbance was measured at 532 nm using a CLARIOstar microplate reader (BMG Labtech, Ortenberg, Germany). MDA concentrations were determined from a standard curve generated with 1,1,3,3-tetramethoxypropane and expressed as nmol/mL, accounting for a two-fold dilution factor. Each sample was analyzed in two independent experiments, with each experiment performed in duplicate. The intra-assay coefficient of variation (CV) was 8.3%, and the inter-assay CV was 5.6%, demonstrating good assay precision. The limit of detection (LOD) and limit of quantification (LOQ) were 0.149 and 0.450 nmol/mL, respectively.

### 2.4 Measurement of serum AOPP levels

Serum AOPP levels were quantified as described in our previous report [19]. Briefly, each assay was conducted in triplicate, with eight serum samples processed per 96-well microtiter plate. Serum samples were diluted 1:10 in phosphate-buffered saline (PBS), and 300 μL of the diluted solution was dispensed into each well of the plate (Corning, Costar, Merck KGaA, Darmstadt, Germany). Chloramine-T hydrate standards (0–100 μM; 300 μL per well) prepared in PBS were included on the same plate, with PBS serving as the blank control. To each well, 15 μL of 1.16 mol/L potassium iodide (Merck KGaA, Darmstadt, Germany) was added, followed by a 2-min incubation at room temperature. Subsequently, 30 μL of glacial acetic acid (Merck KGaA, Darmstadt, Germany) was introduced into all wells. The plate was then centrifuged at 2934×*g* for 5 min and 230 μL of the resulting protein-free supernatant was transferred to unused wells on the same microtiter plate. Absorbance at 340 nm was measured using an Epoch™ microplate spectrophotometer (BioTek Instruments GmbH, Bad Friedrichshall, Germany). Serum AOPP concentrations were calculated from standard curve data using linear regression and are expressed as micromoles per liter (μmol/L) of chloramine-T equivalents. The intra-assay coefficient of variation (CV) was 7.5%, and the inter-assay CV was 5.2%. The LOD and LOQ were 0.890 and 2.696 μmol/L, respectively.

### 2.5 SOD activity assay

SOD activity was measured using a commercial SOD activity assay kit (Cat. No. CS0009, Sigma-Aldrich, St. Louis, MO, USA) in accordance with the manufacturer's instructions. The assay is based on the inhibition of a formazan-producing reaction, in which superoxide anions generated by xanthine oxidase reduce a water-soluble tetrazolium dye to yield a yellow formazan compound, measurable at 450 nm. SOD competes with the dye for superoxide radicals, thus decreasing formazan formation; a reduction in absorbance corresponds to SOD activity. A standard curve was constructed using known concentrations of SOD enzyme. Serum samples were diluted 10-fold prior to analysis. Background absorbance values were determined and subtracted individually for each sample. Absorbance was recorded at 450 nm using a CLARIOstar microplate spectrophotometer (BMG Labtech, Ortenberg, Germany). SOD activity was calculated from the standard curve and expressed as units per milliliter (U/mL). Each sample was analyzed in duplicate, yielding an intra-assay CV of 5.0% and an inter-assay CV of 5.7%. LOD and LOQ were 0.372 and 1.128 U/L, respectively.

### 2.6 Catalase activity assay

Catalase activity was determined using a modified spectrophotometric method based on protocols by Sinha and Hadwan [20,21]. The assay measures the decomposition of hydrogen peroxide (H_2_O_2_) by catalase. Residual H_2_O_2_ reacts with 1.25% potassium dichromate in acetic acid under high-temperature conditions, forming chromic acetate, which absorbs at 570 nm. The concentration of chromic acetate formed is inversely proportional to catalase activity. A standard curve was generated using H_2_O_2_ concentrations ranging from 0 to 200 mM, each reacted with the dichromate reagent. For the assay, serum samples were diluted six-fold in 50 mM phosphate-buffered saline (PBS) and incubated in duplicate with 100 mM H_2_O_2_ to achieve a final concentration of 80 mM. Sample background controls were prepared by substituting PBS for H_2_O_2_. After a 3-min incubation with H_2_O_2_, 1.25% potassium dichromate solution was added, and the mixtures were heated at 95 °C for 10 min using a thermomixer (Thermo Fisher Scientific, Waltham, MA, USA). Samples were then cooled, centrifuged at 10,000×*g* for 5 min at 4 °C, and 200 μL of the supernatant was transferred to a 96-well plate. Absorbance was measured at 570 nm using a CLARIOstar microplate reader (BMG Labtech, Ortenberg, Germany). Catalase activity was calculated from the standard curve after serum background correction and expressed as micromoles of H_2_O_2_ decomposed per minute, or units per milliliter (U/mL). Each sample was analyzed in duplicate, yielding an intra-assay CV of 5.1% and an inter-assay CV of 4.7%. The LOD and LOQ were 2.500 and 7.576 U/L, respectively.

### 2.7 Protein carbonyls

Protein carbonyls were initially evaluated but excluded from final analyses due to levels falling below the LOQ in >33% of samples.

### 2.8 Statistical analyses

Data are presented as medians with interquartile ranges (IQR, q1-q3) or as absolute and relative frequencies, as appropriate. Group comparisons were performed using the Mann–Whitney *U* test or Fisher's exact test. To evaluate the association between oxidative stress markers and mortality outcomes at different time points (in-hospital, 6-month, and 12-month), we performed both univariable and multivariable logistic regression analyses. Additionally, the association between MDA and mortality within 12 months was also evaluated using Cox proportional hazards regression analyses.

Receiver operating characteristic (ROC) curve analyses were used to determine the area under the curve (AUC) and corresponding 95% confidence intervals (CIs) for MDA, N-terminal pro-brain natriuretic peptide (NT-proBNP), and established clinical risk scores. These included the Acute Decompensated Heart Failure National Registry (ADHERE) risk tree [22], the American Heart Association Get With the Guidelines–Heart Failure (GWTG-HF) risk score [23], and the Organized Program to Initiate Lifesaving Treatment in Hospitalized Patients With Heart Failure (OPTIMIZE-HF) risk-prediction algorithm [24]. For internal validation, we also performed Harrell's bootstrap-based method for optimism correction and obtained virtually the same results for the AUCs. In addition, we assessed whether MDA enhances the predictive performance and risk classification based on NT-proBNP and the aforementioned clinical scores. Improvement metrics included changes in AUC and the continuous net reclassification index (cNRI). Furthermore, we performed decision curve analysis (DCA). We compared AUC, cNRI, and the net benefit of models incorporating MDA on top of NT-proBNP or the clinical scores with models containing only NT-proBNP or the clinical scores. We also performed Kaplan-Meier survival analysis for MDA concentrations above vs. below the median and compared them using the log-rank test. Correlations between oxidative stress markers and various clinical and laboratory variables were analyzed using Spearman's rank correlation coefficient. Sensitivity analyses included ROC curve evaluations across various subgroups of patients with AHF, as well as logistic regression analyses with different adjustment models. A two-sided p-value <0.05 was considered statistically significant. All statistical analyses were performed using R software (version 4.4.0).

## 3 Results

### 3.1 Clinical characteristics of the study cohort

A total of 315 patients hospitalized due to AHF were included (median age 76 years; 43.2% female). Most cases (91.4%) presented with worsening chronic HF, and approximately half of those with ischemic origin. Nearly all patients (94.6%) presented with dyspnea at rest (NYHA class IV). Baseline characteristics and chronic medications are summarized in Table 1 and Supplementary Table S1. In-hospital mortality was 10.8% (34 patients), increasing to 31.1% (98 patients) at 6 months and 37.4% (118 patients) at 12 months. Patients who died had more severe HF signs and symptoms at admission, as well as laboratory abnormalities indicative of disease severity, compared with survivors.

**Table 1:** Patient characteristics at hospital admission due to AHF and at different time points according to survival status.

Variable	Alive (N = 281)	In-hospital mortality	P value	Alive (N = 217)	6-month mortality	P value	Alive (N = 197)	12-month mortality	P value	All (N = 315)
		Deceased (N = 34)			Deceased (N = 98)			Deceased (N = 118)		
Demographics										
Age (years)	76.0 (66.0, 81.0)	76.0 (68.8, 86.0)	0.202	73.0 (65.0, 81.0)	79.0 (71.0, 85.0)	**< 0.001**	73.0 (65.0, 81.0)	79.0 (68.2, 85.8)	**<0.001**	76.0 (67.0, 82.0)
Sex, Female	121 (43.1%)	15 (44.1%)	1.000	94 (43.3%)	42 (42.9%)	1.000	85 (43.1%)	51 (43.2%)	1.000	136 (43.2%)
Comorbidities										
Hypertension	264 (94.0%)	30 (88.2%)	0.262	205 (94.5%)	89 (90.8%)	0.232	186 (94.4%)	108 (91.5%)	0.355	294 (93.3%)
T2DM	119 (42.3%)	13 (38.2%)	0.715	86 (39.6%)	46 (46.9%)	0.267	76 (38.6%)	56 (47.5%)	0.127	132 (41.9%)
CAD	142 (50.5%)	14 (41.2%)	0.365	111 (51.2%)	45 (45.9%)	0.397	100 (50.8%)	56 (47.5%)	0.642	156 (49.5%)
CMP	257 (91.5%)	31 (91.2%)	1.000	193 (88.9%)	95 (96.9%)	**0.017**	173 (87.8%)	115 (97.5%)	**0.003**	288 (91.4%)
AF	149 (53.0%)	21 (61.8%)	0.367	110 (50.7%)	60 (61.2%)	0.089	98 (49.7%)	72 (61.0%)	0.062	170 (54.0%)
CKD	121 (43.1%)	22 (64.7%)	**0.018**	81 (37.3%)	62 (63.3%)	**< 0.001**	72 (36.5%)	71 (60.2%)	**<0.001**	143 (45.4%)
MetS	193 (68.7%)	24 (70.6%)	1.000	146 (67.3%)	71 (72.4%)	0.430	130 (66.0%)	87 (73.7%)	0.168	217 (68.9%)
Physical measures at admission										
MAP (mmHg)	101.7 (90.0, 120.0)	90.0 (77.1, 101.2)	**< 0.001**	103.3 (90.0, 123.3)	90.8 (83.3, 106.7)	**< 0.001**	105.0 (90.0, 123.3)	93.3 (83.3, 106.7)	**<0.001**	100.0 (88.3, 118.3)
Heart rate (beats/min)	100.0 (80.0, 117.0)	100.0 (70.8, 114.0)	0.328	100.0 (85.0, 115.0)	95.5 (72.2, 118.8)	0.059	100.0 (85.0, 120.0)	95.0 (72.2, 111.8)	**0.009**	100.0 (80.0, 116.0)
Respiratory rate (breaths/min)	28.0 (24.0, 32.0)	30.0 (26.0, 34.8)	**0.024**	28.0 (24.0, 34.0)	28.0 (24.0, 32.0)	0.816	28.0 (24.0, 34.0)	28.0 (24.0, 32.0)	0.773	28.0 (24.0, 33.0)
BMI (kg/m^2^)	27.9 (25.0, 31.2)	29.9 (26.2, 35.1)	0.058	27.5 (24.8, 31.1)	29.2 (25.4, 32.8)	**0.048**	27.4 (24.9, 30.7)	29.1 (25.3, 32.8)	0.067	28.0 (25.0, 31.6)
Signs and symptoms										
Symptom duration (days)	5.0 (4.0, 5.0)	5.0 (4.0, 5.0)	0.551	5.0 (3.0, 5.0)	5.0 (4.0, 5.0)	0.291	5.0 (3.0, 5.0)	5.0 (4.0, 5.0)	**0.022**	5.0 (4.0, 5.0)
Rales or crackles	277 (98.6%)	34 (100.0%)	1.000	213 (98.2%)	98 (100.0%)	0.314	193 (98.0%)	118 (100.0%)	0.301	311 (98.7%)
JVD	153 (54.4%)	21 (61.8%)	0.469	109 (50.2%)	65 (66.3%)	**0.010**	97 (49.2%)	77 (65.3%)	**0.007**	174 (55.2%)
Enlarged liver	151 (53.7%)	25 (73.5%)	**0.029**	110 (50.7%)	66 (67.3%)	**0.007**	95 (48.2%)	81 (68.6%)	**<0.001**	176 (55.9%)
Ascites	44 (15.7%)	5 (14.7%)	1.000	25 (11.5%)	24 (24.5%)	**0.004**	20 (10.2%)	29 (24.6%)	**0.001**	49 (15.6%)
Peripheral edema	179 (63.7%)	25 (73.5%)	0.342	132 (60.8%)	72 (73.5%)	**0.031**	114 (57.9%)	90 (76.3%)	**<0.001**	204 (64.8%)
NYHA class			0.233			0.105			0.305	
3	17 (6.0%)	0 (0.0%)		15 (6.9%)	2 (2.0%)		13 (6.6%)	4 (3.4%)		17 (5.4%)
4	264 (94.0%)	34 (100.0%)		202 (93.1%)	96 (98.0%)		184 (93.4%)	114 (96.6%)		298 (94.6%)
AHF type			1.000			**0.017**			**0.003**	
New onset AHF	24 (8.5%)	3 (8.8%)		24 (11.1%)	3 (3.1%)		24 (12.2%)	3 (2.5)%		27 (8.6%)
AHF following CHF	257 (91.5%)	31 (91.2%)		193 (88.9%)	95 (96.9%)		173 (87.8%)	115 (97.5%)		288 (91.4%)
Echocardiography										
LVEDd/BSA (mm/m^2^)	28.9 (25.5, 32.0)	27.3 (24.4, 28.3)	**0.038**	29.2 (25.7, 32.1)	27.7 (24.4, 30.6)	0.076	29.3 (25.7, 32.0)	27.8 (24.9, 31.0)	0.234	28.5 (25.5, 31.8)
LVEF (%)	40.0 (30.0, 50.0)	35.0 (25.0, 46.5)	0.316	40.0 (30.0, 50.0)	39.0 (30.0, 48.0)	0.620	40.0 (30.0, 50.0)	39.0 (30.0, 48.0)	0.403	40.0 (30.0, 50.0)
SPAP (mmHg)	50.0 (45.0, 55.0)	55.0 (50.0, 60.0)	**0.010**	45.5 (42.0, 55.0)	55.0 (47.0, 60.0)	**< 0.001**	47.0 (42.0, 55.0)	50.0 (45.0, 60.0)	**0.005**	50.0 (45.0, 60.0)
AHF class			0.755			0.779			0.575	
HFrEF, EF < 40%	128 (46.4%)	15 (55.6%)		98 (45.8%)	45 (50.6%)		88 (44.9%)	55 (51.4%)		143 (47.2%)
HFmrEF, EF 41-49%	75 (27.2%)	6 (22.2%)		59 (27.6%)	22 (24.7%)		55 (28.1%)	26 (24.3%)		81 (26.7%)
HFpEF, EF ≥ 50%	73 (26.4%)	6 (22.2%)		57 (26.6%)	22 (24.7%)		53 (27.3%)	26 (24.3%)		79 (26.1%)
Laboratory test results at admission										
TC (mg/dL)	3.6 (2.9, 4.6)	3.0 (2.7, 4.1)	**0.022**	3.8 (3.1, 4.8)	3.2 (2.6, 4.0)	**< 0.001**	3.8 (3.1, 4.9)	3.3 (2.7, 4.1)	**< 0.001**	3.5 (2.9, 4.5)
HDL-C (mg/dL)	1.1 (0.9, 1.4)	0.9 (0.7, 1.2)	**0.003**	1.1 (0.9, 1.4)	1.0 (0.8, 1.2)	**0.002**	1.1 (0.9, 1.4)	1.1 (0.8, 1.3)	**0.022**	1.1 (0.9, 1.3)
LDL-C (mg/dL)	1.9 (1.4, 2.7)	1.6 (1.3, 2.5)	0.098	2.1 (1.5, 2.8)	1.7 (1.3, 2.3)	**< 0.001**	2.0 (1.5, 2.8)	1.7 (1.3, 2.4)	**< 0.001**	1.9 (1.4, 2.7)
Triglycerides (mg/dL)	1.0 (0.8, 1.3)	1.0 (0.8, 1.3)	0.785	1.0 (0.8, 1.4)	1.0 (0.8, 1.2)	0.193	1.0 (0.8, 1.4)	1.0 (0.8, 1.2)	0.099	1.0 (0.8, 1.3)
Albumin (g/L)	37.9 (35.0, 41.5)	37.3 (34.7, 39.4)	0.173	38.2 (35.3, 42.0)	36.5 (33.9, 39.4)	**0.005**	38.2 (35.5, 42.0)	36.7 (33.8, 39.7)	**0.009**	37.8 (34.8, 41.3)
Total proteins (g/L)	67.0 (61.0, 72.0)	66.0 (61.0, 73.0)	0.972	67.0 (62.0, 72.0)	66.0 (61.0, 71.0)	0.625	67.0 (62.0, 72.0)	65.5 (61.0, 70.0)	0.214	67.0 (61.0, 72.0)
Bilirubin (μmol/L)	17.1 (11.1, 27.5)	21.1 (11.3, 37.0)	0.185	17.4 (11.0, 28.4)	17.2 (11.9, 30.1)	0.332	17.4 (11.0, 28.5)	17.2 (11.9, 29.2)	0.336	17.3 (11.1, 28.7)
AST (U/L)	26.0 (20.0, 42.0)	33.0 (21.5, 161.2)	0.072	28.0 (21.0, 42.0)	26.5 (19.0, 53.8)	0.662	28.0 (22.0, 42.0)	27.0 (18.2, 52.5)	0.542	28.0 (20.0, 44.5)
ALT (U/L)	24.0 (15.0, 40.0)	28.5 (17.0, 164.8)	0.172	25.0 (16.0, 42.0)	21.0 (13.2, 41.5)	0.276	25.0 (16.0, 41.0)	21.0 (14.0, 46.5)	0.226	25.0 (15.0, 42.0)
Glucose (mmol/L)	7.8 (6.0, 11.0)	9.0 (6.8, 11.7)	0.114	7.6 (6.0, 10.7)	8.6 (6.3, 12.3)	0.062	7.7 (6.0, 10.8)	8.1 (6.3, 11.6)	0.267	7.9 (6.1, 11.2)
Sodium (mmol/L)	140.0 (137.0, 142.0)	137.0 (135.0, 140.0)	**0.008**	140.0 (137.0, 142.0)	138.0 (135.0, 141.0)	**< 0.001**	140.0 (138.0, 142.0)	138.0 (135.0, 141.0)	**< 0.001**	140.0 (136.5, 142.0)
Potassium (mmol/L)	4.5 (4.1, 4.8)	4.7 (4.0, 5.1)	0.294	4.5 (4.1, 4.8)	4.5 (4.1, 5.0)	0.611	4.5 (4.1, 4.8)	4.5 (4.1, 5.0)	0.194	4.5 (4.1, 4.8)
Chloride (mmol/L)	103.0 (99.0, 107.0)	98.5 (95.2, 101.0)	**< 0.001**	104.0 (101.0, 107.0)	100.0 (96.2, 103.0)	**< 0.001**	104.0 (101.0, 107.0)	100.0 (97.0, 104.0)	**< 0.001**	103.0 (99.0, 106.0)
BUN (mmol/L)	9.0 (6.8, 13.6)	14.0 (11.2, 18.1)	**< 0.001**	8.4 (6.4, 12.7)	12.3 (8.9, 17.4)	**< 0.001**	8.3 (6.3, 12.3)	12.3 (8.9, 16.8)	**< 0.001**	9.6 (6.9, 14.4)
Creatinine (μmol/L)	114.0 (88.0, 150.0)	133.5 (112.2, 166.0)	**0.015**	110.0 (86.0, 144.0)	136.5 (106.2, 169.5)	**< 0.001**	107.0 (86.0, 144.0)	131.5 (107.0, 164.0)	**< 0.001**	117.0 (90.5, 152.5)
eGFR (ml/min/1.73m^2^)	47.9 (32.7, 67.1)	38.3 (29.5, 49.6)	**0.005**	53.7 (35.6, 69.6)	38.0 (29.2, 50.2)	**< 0.001**	54.0 (36.1, 70.5)	38.4 (29.1, 52.1)	**< 0.001**	46.6 (32.3, 65.0)
CK (U/L)	94.0 (59.0, 163.0)	84.0 (55.0, 193.2)	0.988	98.0 (63.0, 164.0)	79.5 (51.0, 165.8)	**0.043**	105.0 (65.0, 174.0)	78.0 (50.2, 147.5)	**0.007**	93.0 (58.0, 165.5)
LDH (U/L)	259.0 (218.0, 325.0)	298.5 (252.5, 546.8)	**0.014**	252.0 (216.0, 312.0)	289.5 (240.2, 401.8)	**0.002**	252.0 (217.0, 316.0)	283.0 (230.8, 372.2)	**0.029**	265.0 (218.5, 332.0)
hsTnI (ng/L)	41.0 (20.0, 134.5)	120.0 (39.8, 265.0)	**0.009**	39.0 (17.0, 128.5)	67.0 (31.0, 161.0)	**0.004**	39.0 (17.5, 136.5)	61.0 (30.0, 149.0)	**0.039**	46.0 (20.0, 143.2)
NT-proBNP (pg/mL)	6112.0 (3386.0, 12742.0)	13076.0 (6461.0, 21952.2)	**< 0.001**	5466.0 (3151.0, 11515.0)	10810.5 (5855.0, 19095.5)	**< 0.001**	5350.0 (3151.0, 10691.0)	10733.0 (5486.5, 18385.5)	**< 0.001**	6692.0 (3531.0, 14395.5)
CRP (mg/L)	11.4 (5.3, 29.6)	30.4 (10.1, 57.0)	**0.001**	10.2 (4.6, 23.0)	26.9 (8.0, 52.9)	**< 0.001**	10.3 (4.9, 21.9)	24.9 (6.4, 47.3)	**< 0.001**	12.2 (5.5, 33.1)
IL-6 (pg/mL)	24.5 (12.4, 54.3)	66.8 (22.2, 133.0)	**< 0.001**	21.5 (11.3, 43.9)	49.1 (19.4, 87.1)	**< 0.001**	22.1 (11.3, 44.8)	40.6 (17.1, 79.6)	**< 0.001**	25.1 (12.9, 60.1)
Fibrinogen (g/L)	4.0 (3.4, 4.7)	4.0 (3.0, 5.0)	0.974	4.0 (3.5, 4.7)	4.0 (3.0, 4.9)	0.565	4.0 (3.4, 4.7)	4.0 (3.1, 4.9)	0.469	4.0 (3.4, 4.8)
Erythrocytes (x 10^12^/L)	4.6 (4.2, 5.1)	4.3 (3.8, 4.9)	**0.044**	4.7 (4.3, 5.1)	4.3 (3.7, 4.9)	**< 0.001**	4.7 (4.4, 5.1)	4.4 (3.8, 4.9)	**< 0.001**	4.6 (4.2, 5.1)
Hemoglobin (g/L)	135.0 (120.0, 148.0)	122.0 (109.5, 139.5)	**0.008**	138.0 (124.0, 149.0)	123.0 (109.0, 139.0)	**< 0.001**	138.0 (124.0, 150.0)	126.0 (111.0, 141.0)	**< 0.001**	134.0 (119.0, 148.0)
Leukocytes (x10^12^/L)	9.6 (7.4, 13.1)	10.8 (8.0, 13.4)	0.337	9.4 (7.1, 12.6)	10.7 (7.8, 13.6)	0.185	9.5 (7.2, 12.6)	10.5 (7.6, 13.6)	0.311	9.9 (7.5, 13.1)
Platelets (x10^9^/L)	221 (181, 272)	232 (187, 317)	0.323	224 (184, 270)	221 (173, 299)	0.941	224 (184, 270)	223 (173, 284)	0.921	224 (181, 277)
pH	7.4 (7.3, 7.4)	7.4 (7.3, 7.5)	0.756	7.4 (7.4, 7.5)	7.4 (7.3, 7.4)	0.254	7.4 (7.3, 7.5)	7.4 (7.3, 7.4)	0.709	7.4 (7.3, 7.5)
pO_2_ (kPa)	8.8 (7.2, 10.4)	8.6 (7.4, 11.4)	0.842	8.9 (7.2, 10.4)	8.7 (7.3, 10.5)	0.754	8.8 (7.2, 10.4)	8.8 (7.3, 10.4)	0.803	8.8 (7.2, 10.4)
pCO_2_ (kPa)	5.2 (4.5, 6.3)	5.4 (4.2, 7.5)	0.635	5.2 (4.4, 6.3)	5.4 (4.6, 7.2)	0.131	5.2 (4.4, 6.3)	5.2 (4.5, 7.1)	0.386	5.2 (4.5, 6.4)
HCO_3_ (mmol/L)	23.9 (21.3, 27.4)	24.6 (20.1, 27.6)	0.844	23.9 (21.2, 27.1)	24.2 (21.3, 28.7)	0.489	23.9 (21.2, 27.0)	24.4 (21.3, 28.9)	0.368	23.9 (21.3, 27.4)

Data are presented as n (%) or medians (q1, q3). Differences between AHF patients who survived and those who died in-hospital or within 6 or 12 months after index AHF hospitalization were tested with the Fisher's exact test or Mann-Whitney *U* test. P-values <0.05 are considered significant and are depicted in bold.

AF, atrial fibrillation; AHF, acute heart failure; ALT, alanine aminotransferase; AST, aspartate aminotransferase; BMI, body mass index; BUN, blood urea nitrogen; CAD, coronary artery disease; CHF, chronic heart failure; CK, creatine kinase; CKD, chronic kidney disease; CMP, cardiomyopathy; CRP, C-reactive protein; EF, ejection fraction; eGFR, estimated glomerular filtration rate; HDL-C, high-density lipoprotein cholesterol; HFmrEF, heart failure with mildly reduced ejection fraction; HFpEF, heart failure with preserved ejection fraction; HFrEF, heart failure with reduced ejection fraction; hsTnI, high-sensitivity troponin I; IL-6, interleukin 6; JVD, jugular vein distension; LDH, lactate dehydrogenase; LDL-C, low-density lipoprotein cholesterol; LVEDd, left ventricle end-diastolic diameter; LVEF, left ventricular ejection fraction; MAP, mean arterial pressure; MetS, metabolic syndrome; NT-proBNP, N-terminal pro brain natriuretic peptide; NYHA, New York Heart Association; pO_2_, partial oxygen pressure; pCO2, partial carbon dioxide pressure; SPAP, systolic pulmonary artery pressure; TC, total cholesterol; T2DM, diabetes mellitus type 2.

Missing data: AHF class – data for 12 participants.

### 3.2 Association between indicators of oxidative stress and mortality in AHF patients

**Fig. 1:**
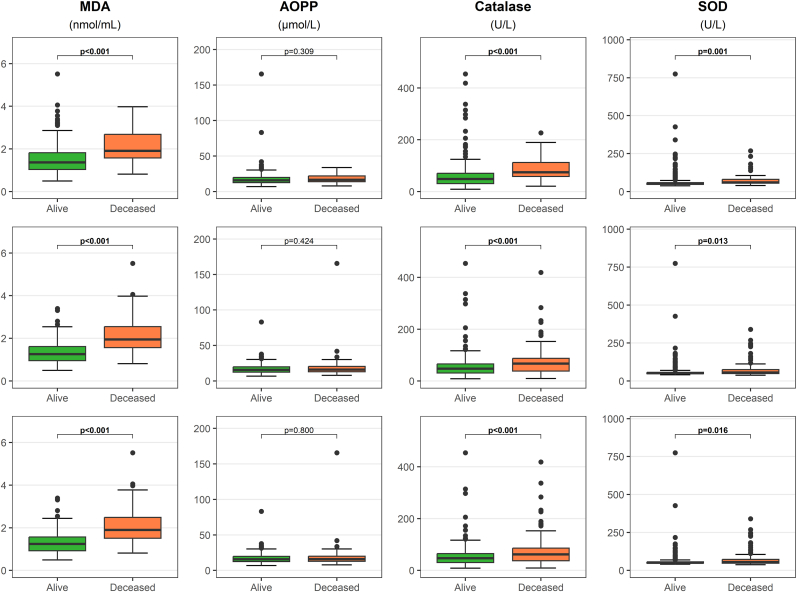
Comparison of oxidative stress marker levels between patients who survived and those who died in-hospital, as well as at 6 and 12 months following index AHF hospitalization. Group differences were assessed using the Mann–Whitney *U* test. For MDA and SOD 314 samples and for catalase 304 samples were available for this and subsequent analyses. P-values <0.05 were considered statistically significant. AOPP, advanced oxidation protein products; MDA, malondialdehyde; SOD, superoxide dismutase.

**Fig. 2:**
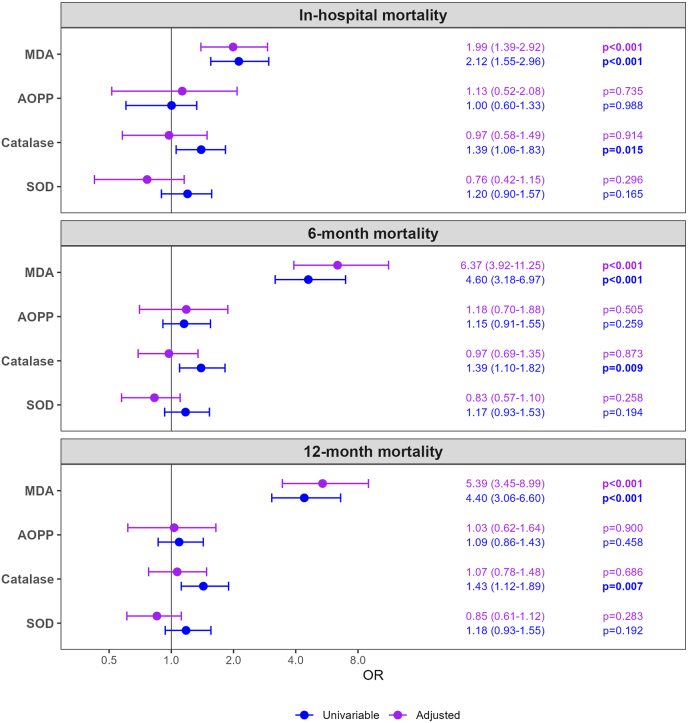
Univariable and multivariable logistic regression analyses of oxidative stress parameters as predictors of in-hospital, 6-month, and 12-month mortality in patients with AHF. Results are presented as OR and 95% CI per 1-SD increase. Multivariable model was adjusted for age, sex, BMI, NT-proBNP, MAP, eGFR, BUN, ALT, albumin, and IL-6. P-values <0.05 were considered statistically significant. ALT, alanine aminotransferase; AOPP, advanced oxidation protein products; BMI, body mass index; BUN, blood urea nitrogen; CI, confidence interval; eGFR, estimated glomerular filtration rate; IL-6, interleukin-6; MAP, mean arterial pressure; MDA, malondialdehyde; NT-proBNP, N-terminal pro brain natriuretic peptide; OR, odds ratio; SD, standard deviation; SOD, superoxide dismutase.

Serum MDA, catalase, and SOD levels at admission were higher in patients who died during hospitalization or within 6 or 12 months, whereas AOPPs showed no significant differences (Fig. 1). In univariable analyses, MDA and catalase, but not SOD, were associated with mortality (Fig. 2). After adjustment for clinical and laboratory variables, only MDA remained independently associated with mortality across all models (Fig. 2 and Supplementary Table S2), highlighting its robust prognostic value in patients with AHF. Additional Cox regression analyses yielded similar results, with higher baseline MDA levels remaining significantly associated with mortality risk (Supplementary Fig. S1).

### 3.3 Prognostic ability and clinical utility of MDA

**Fig. 3:**
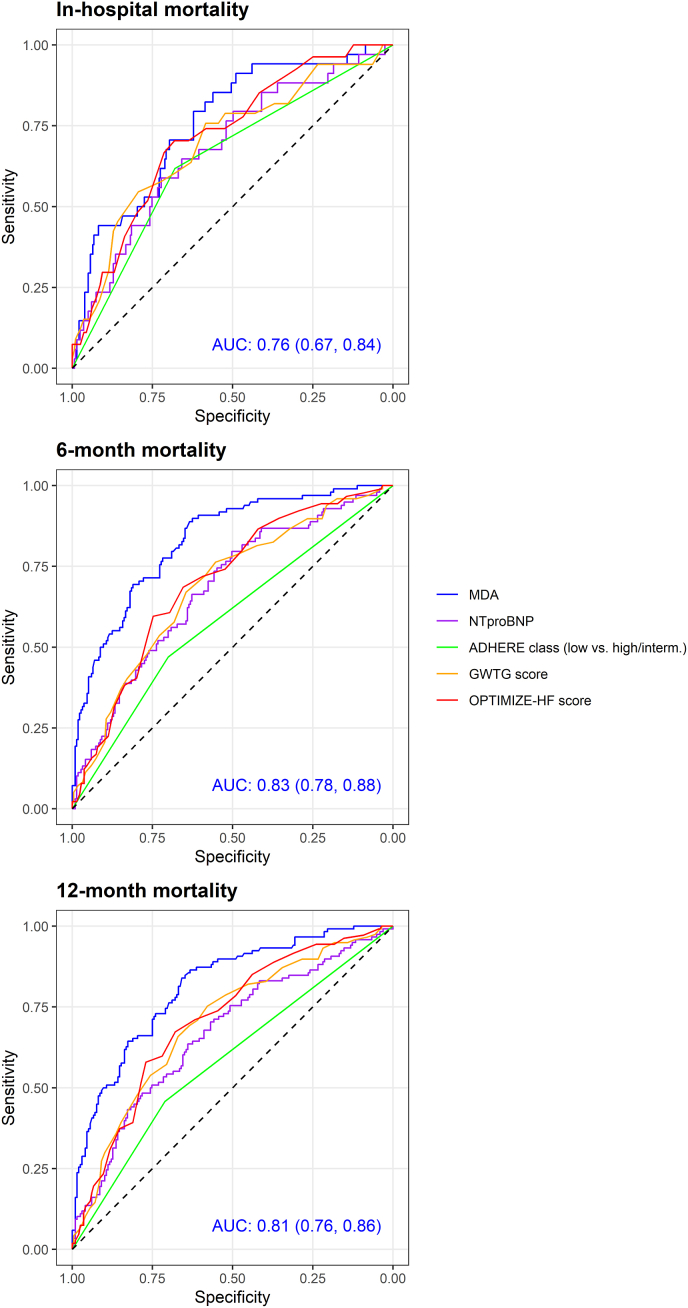
Receiver operating characteristic curves for prediction of in-hospital, 6-month, and 12-month mortality using MDA, NT-proBNP, ADHERE class, GWTG score, and OPTIMIZE-HF score. AUC, area under the curve; CI, confidence interval; ADHERE, Acute Decompensated Heart Failure National Registry; GWTG-HF, Get With the Guidelines-Heart Failure score; MDA, malondialdehyde; NT-proBNP, N-terminal pro-brain natriuretic peptide; OPTIMIZE-HF, Organized Program to Initiate Lifesaving Treatment in Hospitalized Patients With Heart Failure.

**Fig. 4:**
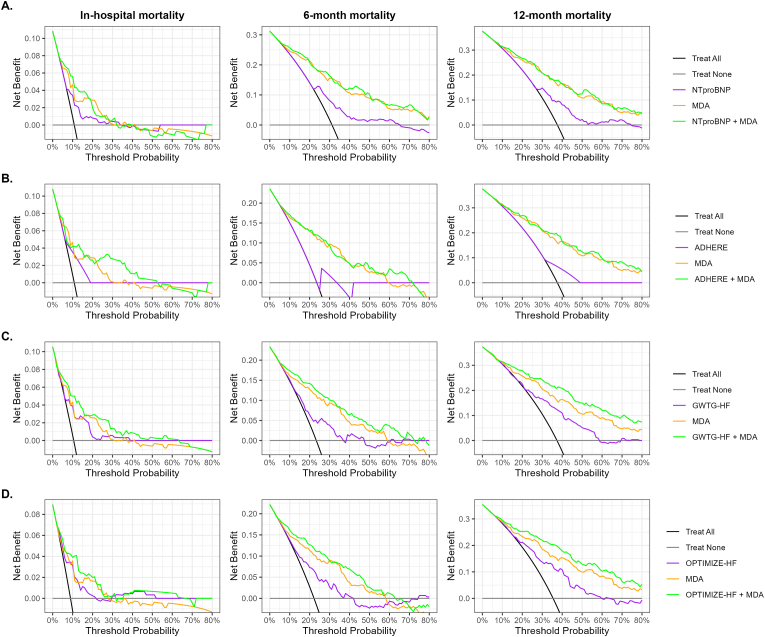
Decision curve analysis comparing net benefit of baseline NT-proBNP or risk scores alone with models also containing MDA for predicting in-hospital, 6-month, and 12-month mortality of patients with AHF. ADHERE, Acute Decompensated Heart Failure National Registry; AHF, acute heart failure; GWTG-HF, Get With The Guidelines Heart Failure; MDA, malondialdehyde; NT-proBNP, N-terminal pro-brain natriuretic peptide; OPTIMIZE-HF, Organized Program to Initiate Lifesaving Treatment in Hospitalized Patients With Heart Failure.

**Fig. 5:**
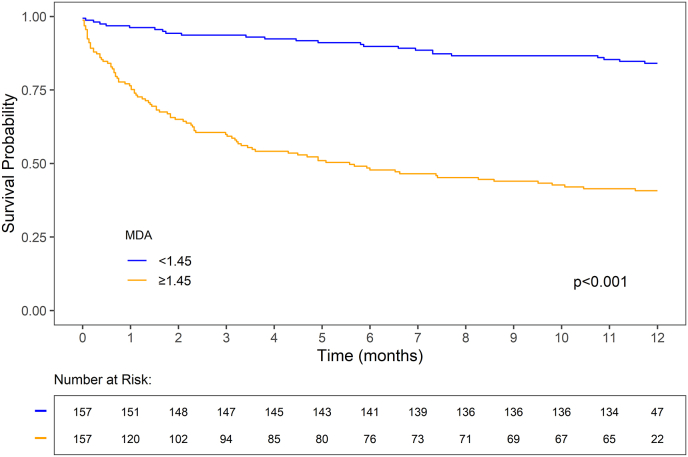
Kaplan–Meier survival curves showing 12-month mortality in patients with AHF stratified by MDA levels. The number of patients at risk at each time point is shown below the graph. AHF, acute heart failure; MDA, malondialdehyde.

ROC curve analyses showed that MDA had higher AUCs than NT-proBNP and clinical scores for predicting in-hospital, 6-month, and 12-month mortality, with particularly high accuracy for longer-term outcomes (Fig. 3, Supplementary Table S3). Its clinical utility was further supported by significant improvements in AUC, cNRI, and net benefit when added to NT-proBNP or clinical scores (Table 2, Fig. 4). Kaplan–Meier analysis confirmed that patients with MDA levels below the median (1.45 nmol/L) had significantly higher survival than those with higher levels (p < 0.001, Fig. 5).

**Table 2:** Improvement in discrimination and classification by MDA for predicting in-hospital, 6-month, and 12-month mortality.

	AUC (95% CI)	ΔAUC (95% CI)	p-value	cNRI (95% CI)	p-value
**In-hospital mortality**					
**NT-proBNP**	0.68 (0.58-0.78)	Reference			
+MDA	0.80 (0.73-0.88)	0.12 (0.01-0.23)	**0.026**	0.47 (0.12-0.83)	**0.008**
**ADHERE**	0.65 (0.56-0.74)	Reference			
+ MDA	0.76 (0.67-0.85)	0.11 (0.05-0.17)	**<0.001**	0.53 (0.18-0.88)	**0.003**
**GWTG-HF**	0.70 (0.60-0.80)	Reference			
+ MDA	0.80 (0.72-0.88)	0.10 (0.03-0.17)	**0.007**	0.58 (0.22-0.93)	**0.001**
**OPTIMIZE-HF**	0.72 (0.62-0.81)	Reference			
+ MDA	0.79 (0.69-0.88)	0.07 (0.01-0.13)	**0.025**	0.49 (0.10-0.88)	**0.014**
**6-month mortalit**y					
**NT-proBNP**	0.68 (0.62-0.74)	Reference			
+MDA	0.84 (0.80-0.89)	0.16 (0.10-0.23)	**<0.001**	0.96 (0.75-1.17)	**<0.001**
**ADHERE**	0.59 (0.53-0.64)	Reference			
+ MDA	0.84 (0.79-0.89)	0.25 (0.19.0.32)	**<0.001**	0.95 (0.73-1.16)	**<0.001**
**GWTG-HF**	0.69 (0.62-0.75)	Reference			
+ MDA	0.86 (0.82-0.90)	0.17 (0.111-0.23)	**<0.001**	0.98 (0.77-1.19)	**<0.001**
**OPTIMIZE-HF**	0.70 (0.64-0.76)	Reference			
+ MDA	0.86 (0.82-0.91)	0.16 (0.10-0.21)	**<0.001**	1.02 (0.80-1.23)	**<0.001**
**12-month mortality**					
**NT-proBNP**	0.67 (0.61-0.73)	Reference			
+MDA	0.82 (0.78-0.87)	0.16 (0.09-0.22)	**<0.001**	0.89 (0.68-1.09)	**<0.001**
**ADHERE**	0.59 (0.53-0.64)	Reference			
+ MDA	0.83 (0.78-0.87)	0.24 (0.18-0.30)	**<0.001**	0.91 (0.70-1.11)	**<0.001**
**GWTG-HF**	0.70 (0.64-0.76)	Reference			
+ MDA	0.86 (0.81-0.90)	0.15 (0.10-0.21)	**<0.001**	1.03 (0.84-1.23)	**<0.001**
**OPTIMIZE-HF**	0.71 (0.65-0.77)	Reference			
+ MDA	0.85 (0.81-0.90)	0.14 (0.009-0.19)	**<0.001**	0.93 (0.72-1.14)	**<0.001**

The ΔAUC indicates the difference between the AUC of the reference model and the model additionally including MDA. *P* values for ΔAUCs were obtained by DeLong's method, while p-values for cNRIs were calculated from z-scores. ADHERE, Acute Decompensated Heart Failure National Registry; AUC, area under the curve; cNRI, continuous net reclassification improvement; GWTG-HF, Get With The Guidelines Heart Failure; MDA, malondialdehyde; NT-proBNP, N-terminal pro brain natriuretic peptide OPTIMIZE-HF, Organized program to Initiate Lifesaving Treatment in Hospitalized Patients With Heart Failure.

## 4 Discussion

Several previous studies have investigated the association between markers of oxidative stress and both the severity of HF [11,[25], [26], [27], [28], [29], [30], [31], [32], [33]] and clinical outcomes in patients with chronic HF [[9], [10], [11], [12], [13]]. However, none have specifically evaluated the prognostic value of oxidative stress biomarkers, including MDA, in patients with AHF. In the present study, we demonstrate for the first time the robust prognostic potential of baseline serum MDA levels for in-hospital, 6-month, and 12-month mortality in patients with AHF. Notably, the addition of MDA to NT-proBNP, a widely used biomarker in HF [34], and to established clinical risk scores, including ADHERE, GWTG-HF, and OPTIMIZE-HF [[22], [23], [24]], improved prognostic discrimination for mortality.

These findings underscore the ability of MDA, a key end-product of lipid peroxidation, to sensitively reflect the complex pathophysiology of AHF. This condition is characterized by hemodynamic impairment, tissue hypoperfusion and congestion, activation of the renin–angiotensin–aldosterone system, increased sympathetic tone, elevated levels of natriuretic peptides, low-grade chronic inflammation, and insulin resistance [2,14,35]. These processes create a highly pro-oxidative environment, ultimately leading to lipid peroxidation in cell membranes and circulating lipoproteins, elevated MDA levels, tissue and organ injury, worsening of HF symptoms, and adverse patient outcome [3,6,7,36].

In the present study, MDA levels showed positive correlations with NT-proBNP, inflammatory mediators such as C-reactive protein and IL-6, and leukocyte counts (Supplementary Fig. S2), consistent with the known interplay between inflammation and oxidative stress in HF [37]. Additionally, MDA levels were higher in AHF patients with metabolic syndrome and type 2 diabetes (Supplementary Table S4), and positively associated with serum glucose levels (Supplementary Fig. S2), in agreement with prior evidence of increased lipid peroxidation in these metabolic states [[38], [39], [40]]. Furthermore, the positive association between MDA concentrations and platelet counts (Supplementary Fig. S2) may indicate a contribution of platelets to MDA generation [40]; however, the higher MDA levels observed in AHF patients receiving chronic acetylsalicylic acid therapy (Supplementary Table S5) argue against platelets being a major source of MDA.

Further supporting the link between oxidative stress and HF pathophysiology, MDA levels were also elevated in patients with chronic kidney disease (Supplementary Table S4), and negatively correlated with eGFR (Supplementary Fig. 2), likely reflecting the pro-inflammatory and pro-oxidative effects of uremic toxins, impaired renal perfusion, congestion, and inflammation [2,35].

Contrary to our findings, a study in patients with chronic HF reported a negative association between MDA and HF severity [41], which the authors attributed to potential confounding effects of medications with antioxidant properties, such as ACE inhibitors, statins, and beta-blockers [[42], [43], [44]]. In our study, however, these medications did not significantly influence MDA levels (Supplementary Table S5).

Interestingly, in the present study, serum catalase and SOD levels were elevated in patients with more severe HF and correlated positively with markers of disease severity (NT-proBNP, BUN) and hepatic injury (ALT, AST) (Supplementary Fig. S2). Together with their higher levels in patients exhibiting venous volume overload (Supplementary Table S4) and prior evidence of their release from damaged tissues [[45], [46], [47], [48]], these findings suggest that the increased circulating catalase and SOD arise primarily from passive leakage following oxidative or mechanical injury, rather than from an adaptive upregulation of systemic antioxidant defenses. In the present study, catalase, SOD, and AOPPs showed only weak associations with mortality, suggesting limited prognostic utility in AHF.

Oxidative stress is a recognized driver of HF progression and is largely mediated by mitochondrial dysfunction and overproduction of reactive oxygen species (ROS) [3,6,36]. Enzymes such as NADPH oxidase, xanthine oxidase, endothelial nitric oxide synthase, cyclooxygenases, lipoxygenases, and myeloperoxidase also contribute to ROS generation, further damaging cardiac and other tissues [3,6,36]. In addition to excessive ROS generation, depletion of endogenous antioxidant systems, such as catalase and SOD, worsens oxidative stress [3,6].

The disruption of redox homeostasis is a key pathogenic mechanism implicated in a wide range of diseases, including cardiovascular disorders, metabolic syndrome, and cancer [4,49,50]. In our study, higher MDA levels in non-survivors likely indicate disrupted redox homeostasis, resulting from an imbalance between pro-oxidant and antioxidant mechanisms.

The increased MDA may actively contribute to myocardial injury through multiple interrelated pathways [4]. Excessive lipid peroxidation can aggravate mitochondrial dysfunction by compromising membrane integrity and impairing electron transport chain efficiency, resulting in reduced ATP synthesis and enhanced ROS leakage [3,51]. Beyond mitochondrial impairment, MDA readily forms covalent adducts with proteins, modifying key structural and signaling molecules involved in excitation–contraction coupling, calcium handling, and extracellular matrix remodeling [3,6,[52], [53], [54]]. Elevated MDA has been reported to depress cardiac contractile function in ventricular myocytes and to exert deleterious effects on coronary endothelial cells by promoting cytotoxicity and disrupting the endothelial glycocalyx [[52], [53], [54]].

Further investigation is needed to determine whether MDA, through upregulation of NADPH oxidases, particularly NOX2 and NOX4, establishes a self-sustaining cycle of ROS generation and tissue injury [53]. Nevertheless, it remains unclear whether MDA directly drives myocardial damage or primarily serves as an integrated marker of systemic oxidative stress in HF. While experimental models indicate cytotoxic and pro-inflammatory properties of MDA adducts, clinical data largely support its interpretation as a cumulative index of oxidative stress burden [10,36,55,56]. Future mechanistic and interventional studies are warranted to elucidate whether MDA represents a causal mediator or a surrogate indicator of oxidative myocardial injury.

Although oxidative stress plays a well-established pathophysiological role in HF, antioxidant therapies that have demonstrated efficacy in animal models largely failed to improve clinical outcomes in human HF trials [3,36]. Nevertheless, our findings highlight a clear and clinically relevant association between oxidative stress, especially lipid peroxidation and poor outcomes in AHF.

### 4.1 Study strengths and limitations

Key strengths of our study include a well-characterized patient cohort and the implementation of a rigorous protocol for serum collection, processing, and storage. Assays were performed under stringent conditions with low intra- and inter-assay variability, contributing to the reliability of the biomarker measurements. However, several limitations should be acknowledged: A major limitation of the present study is that lipid peroxidation and degradation of peroxidation products may occur even at −80 °C. Consequently, prolonged cryopreservation of our serum samples (up to six years) may have affected MDA stability, introducing pre-analytical variability that cannot be fully excluded, and could have influenced the measured concentrations independent of AHF pathophysiology. Given the limited specificity of the TBARS assay employed for MDA quantification, owing to the ability of TBA to react with reactive carbonyl groups from various substances in addition to MDA, the values reported here may reflect a mixture of MDA and other TBA-reactive compounds. Furthermore, because the MDA standard in this study was prepared in water rather than in serum, the absolute MDA concentrations may be affected by matrix effects and should be interpreted as relative indices of systemic oxidative stress. Nevertheless, the MDA concentrations measured in this study are highly consistent with those reported in previous investigations [40,55]. Although our logistic and Cox regression models adjusted for multiple relevant clinical and laboratory parameters, certain unmeasured or unrecorded factors may have influenced oxidative stress levels and the observed association between MDA and mortality. In particular, due to the lack of data on free hemoglobin or a hemolysis index, we could not assess the potential contribution of free hemoglobin to lipid peroxidation and serum MDA levels, or the impact of erythrocyte leakage on SOD and catalase activities. Therefore, potential confounding by hemolysis cannot be excluded. This study was conducted within a single population, which may limit the generalizability of the findings to other populations with differing demographic, ethnic, or clinical characteristics, as well as those within different healthcare systems or practice settings. As such, the applicability of these results to broader or more diverse cohorts remains uncertain. Therefore, future studies involving multi-center and multi-ethnic cohorts are warranted to validate our findings and confirm their robustness across varying clinical environments. The observational nature of the study precludes conclusions regarding causality or underlying mechanisms.

## 5 Conclusions

In conclusion, serum MDA is a predictor of mortality in AHF and enhances the prognostic accuracy of established clinical scores and biomarkers. Given its pathophysiological relevance and predictive value, MDA may serve as a valuable adjunct for risk stratification in clinical practice. Routine assessment of MDA could help identify high-risk patients early and guide timely, targeted interventions to reduce AHF-related mortality. Future studies should focus on validating these findings in larger, independent AHF cohorts, evaluating whether MDA-guided management strategies can improve clinical outcomes.

## Declaration of generative AI and AI-assisted technologies in the manuscript preparation process

During the preparation of this work the authors used ChatGPT (OpenAI) in order to improve readability and fluency of the English language. After using this tool, the authors reviewed and edited the content as needed and take full responsibility for the content of the published article.

## Funding

T.M. is grateful to the Austrian Science Fund (10.13039/501100002428FWF) for excellence cluster 10.55776/COE14, Grants 10.13039/100000201DOI 10.55776/P28854, 10.5576/I3792, 10.55776/DOC130, and 10.55776/W1226, the 10.13039/501100004955Austrian Research Promotion Agency (10.13039/501100004955FFG) grants 864690 and 870454, the Integrative Metabolism Research Center Graz, the Austrian 10.13039/100031425Infrastructure Program 2016/2017, the Styrian Government, the City of Graz and 10.13039/501100019802BioTechMed-Graz. This project was also funded in part by the 10.13039/501100004955FFG and the 10.13039/501100000780European Union (10.13039/501100008530EFRE) under grant 912192. Marija Pinterić was supported by the 10.13039/501100004488Croatian Science Foundation and funded by the 10.13039/501100000780European Union – NextGenerationEU.

The funders had no role in study design, in the collection, analysis and interpretation of data, as well as in the writing of the report and in the decision to submit the article for publication. For open access purposes, the author has applied a CC BY public copyright license to any author accepted manuscript version arising from this submission.

## CRediT authorship contribution statement

**Marija Pinterić:** Data curation, Investigation, Methodology, Validation, Writing – review & editing. **Iva Klobučar:** Conceptualization, Investigation, Visualization, Writing – review & editing. **Margarete Lechleitner:** Investigation, Methodology, Writing – review & editing. **Lidija Hofmann:** Investigation, Methodology, Writing – review & editing. **Matias Trbušić:** Resources, Supervision, Writing – review & editing. **Gudrun Pregartner:** Formal analysis, Visualization, Writing – review & editing. **Andrea Berghold:** Formal analysis, Supervision, Writing – review & editing. **Tobias Madl:** Data curation, Funding acquisition, Writing – review & editing. **Saša Frank:** Conceptualization, Project administration, Supervision, Writing – original draft, Writing – review & editing. **Vesna Degoricija:** Conceptualization, Resources, Supervision, Writing – review & editing.

## Declaration of competing interest

The authors declare the following financial interests/personal relationships which may be considered as potential competing interests: Tobias Madl reports financial support was provided by Austrian Science Fund. Tobias Madl reports financial support was provided by Austrian Research Promotion Agency. Marija Pinterić reports financial support was provided by Croatian Science Foundation. If there are other authors, they declare that they have no known competing financial interests or personal relationships that could have appeared to influence the work reported in this paper.

## Data Availability

Data will be made available on request.
